# Supplementary figures and images for: Characterization of the Sensory Properties and Quality Components of Huangjin Green Tea Based on Molecular Sensory-Omics
Source: Foods. 2023 Aug 28;12(17):3234. doi: 10.3390/foods12173234 (PMC10486783; doi:10.3390/foods12173234)

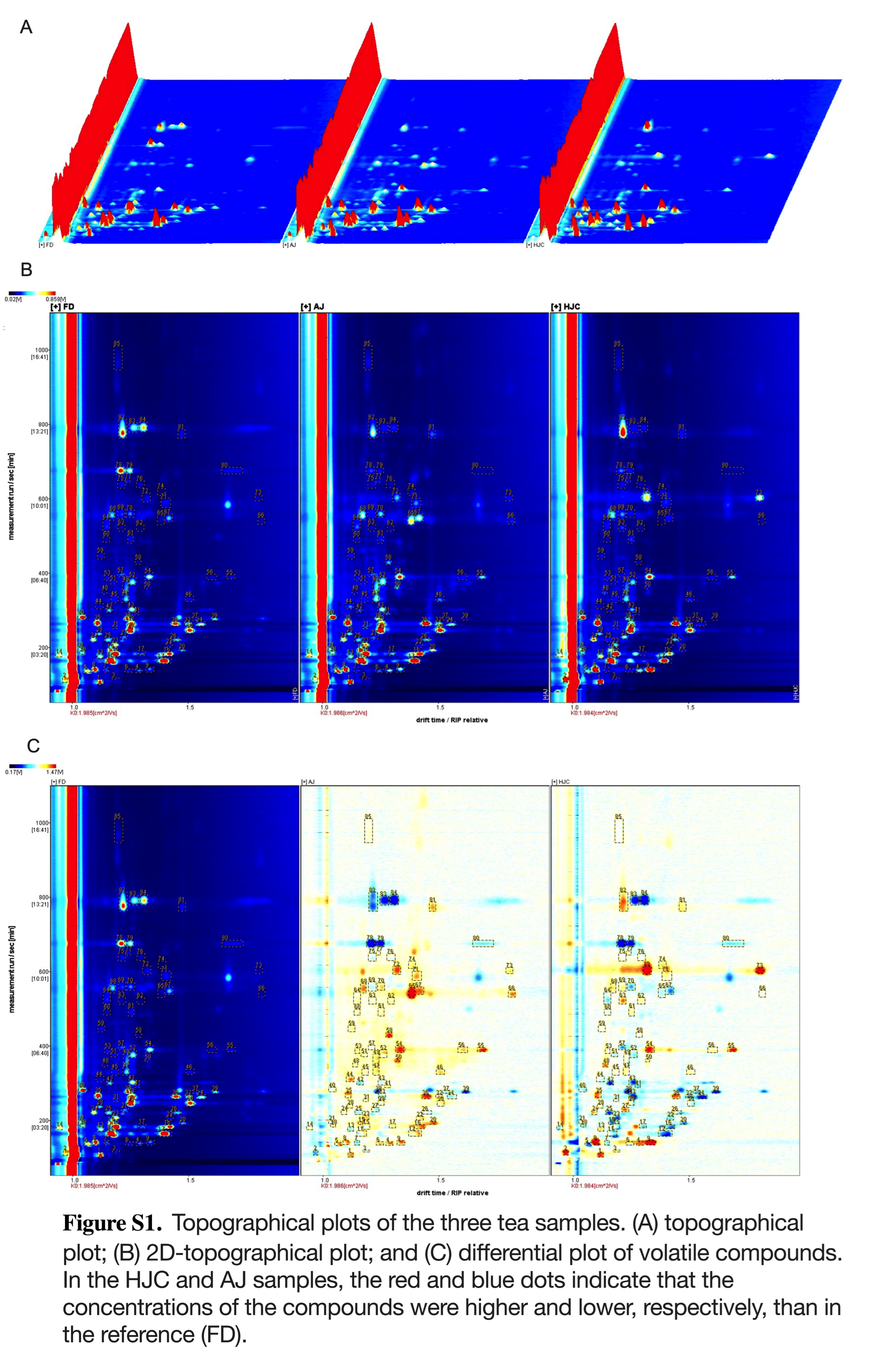

Supplement: Supplementary file 1 [file foods-12-03234-s001.zip › Figure S1.tif]
